# Supplementary material for: Defactinib inhibits PYK2 phosphorylation of IRF5 and reduces intestinal inflammation
Source: Nat Commun. 2021 Nov 18;12:6702. doi: 10.1038/s41467-021-27038-5 (PMC8602323; doi:10.1038/s41467-021-27038-5)
Supplement: Supplementary file 1 — Supplementary Information [file 41467_2021_27038_MOESM1_ESM.pdf]

Defactinib inhibits PYK2 phosphorylation of IRF5 and reduces intestinal inflammation

Grigory Ryzhakov, Hannah Almuttaqi, Alastair L. Corbin, Dorothée L. Berthold, Tariq Khoyratty, Hayley L Eames, Samuel Bullers, Claire Pearson, Zhichao Ai, Kristina Zec, Sarah Bonham, Roman Fischer, Luke Jostins-Dean, Simon P.L. Travis, Benedikt M. Kessler and Irina A. Udalova

Supplementary Information

Supplementary Figure 1

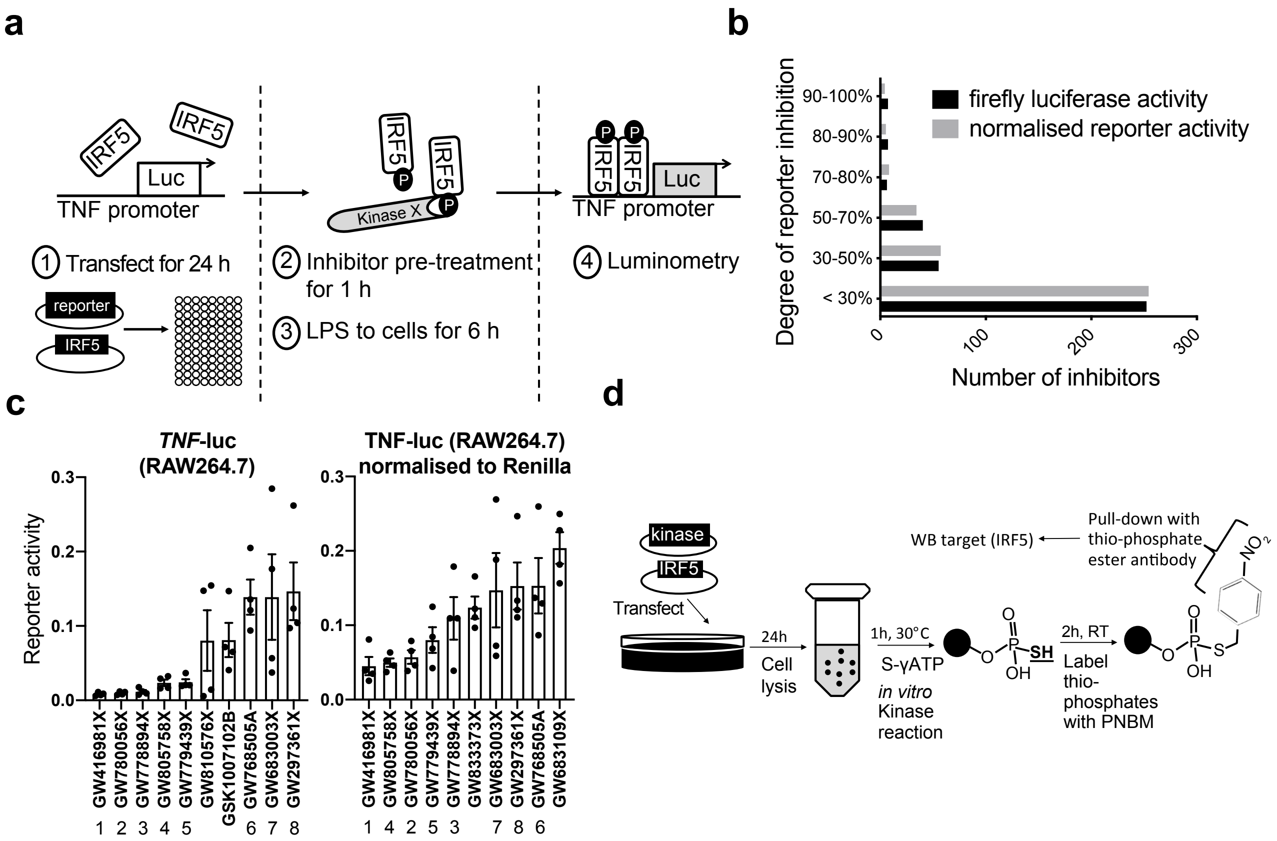

e

KINASE

	ARK5	CLK2	HIPK4	JNK2	MARK3	MST1	PYK2
TNF-luciferase (overexpression)	X	✓	✓	✓	X	✓	✓
IRF5 binding (overexpression)	✓	X	✓	✓	X	X	✓
In vitro kinase assay (overexpression)	X	X	X	✓	✓	✓	✓
IBD locus	X	X	X	X	X	X	✓

Supplementary Fig. 1. Screening and validation assays to identify novel IRF5 kinases. (a) A scheme of small molecule screening for candidate IRF5 kinases. RAW cells were transfected with plasmids encoding for IRF5 and TNF-luciferase reporter as well as constitutively expressed Renilla luciferase. (1) 24 hrs after transfection cells were pre-treated with a library of inhibitors (four replicate wells per inhibitor) for 1hr (2) and stimulated with 1 μg/mL of LPS for 6 hrs (3) before lysing cells for luminometry. **(b)** Stratification of the kinase inhibitors used in the screen based on the degree of IRF5 reporter inhibition. The numbers are shown based on activities of the firefly luciferase reporter, raw values or normalised to Renilla luciferase activities to account for non-specific impact of cell viability. Out of 365 molecules, 57 inhibited IRF5 reporter activity by 30-50%, 34 – 50-70%, 8 – 70-80%, 5 – 80-90% and 4 by >90% (the normalised activity readout). **(c)** Activities of top 10 IRF5 reporter inhibitors are shown where the dataset was analysed based on raw firefly luciferase or normalised to Renilla values. The compounds indicated with numbers are in top 10 independently of normalisation. To calculate reporter activity luciferase values (raw or normalised to Renilla) in wells incubated with kinase inhibitors were divided by the luciferase activity values in the control wells (DMSO vehicle only, cells expressing IRF5 and stimulated with LPS). Data presented as mean values ±SEM from n=4 independent experiments. **(d)** A scheme of a modified-ATP based IRF5 kinase assay. Cells co-expressing HA-tagged IRF5 with either of the candidate kinases were lysed and incubated with S-γ-ATP. The newly-produced phosphate groups were further labelled using a reaction with PNBM and the modified proteins were pulled down using anti-thiophosphate ester antibody. Diagram drawn by authors. **(e)** Table summarising functional validation of candidate kinases related to Fig. 1b-d. Source data are provided as a Source Data file.

Supplementary Figure 2

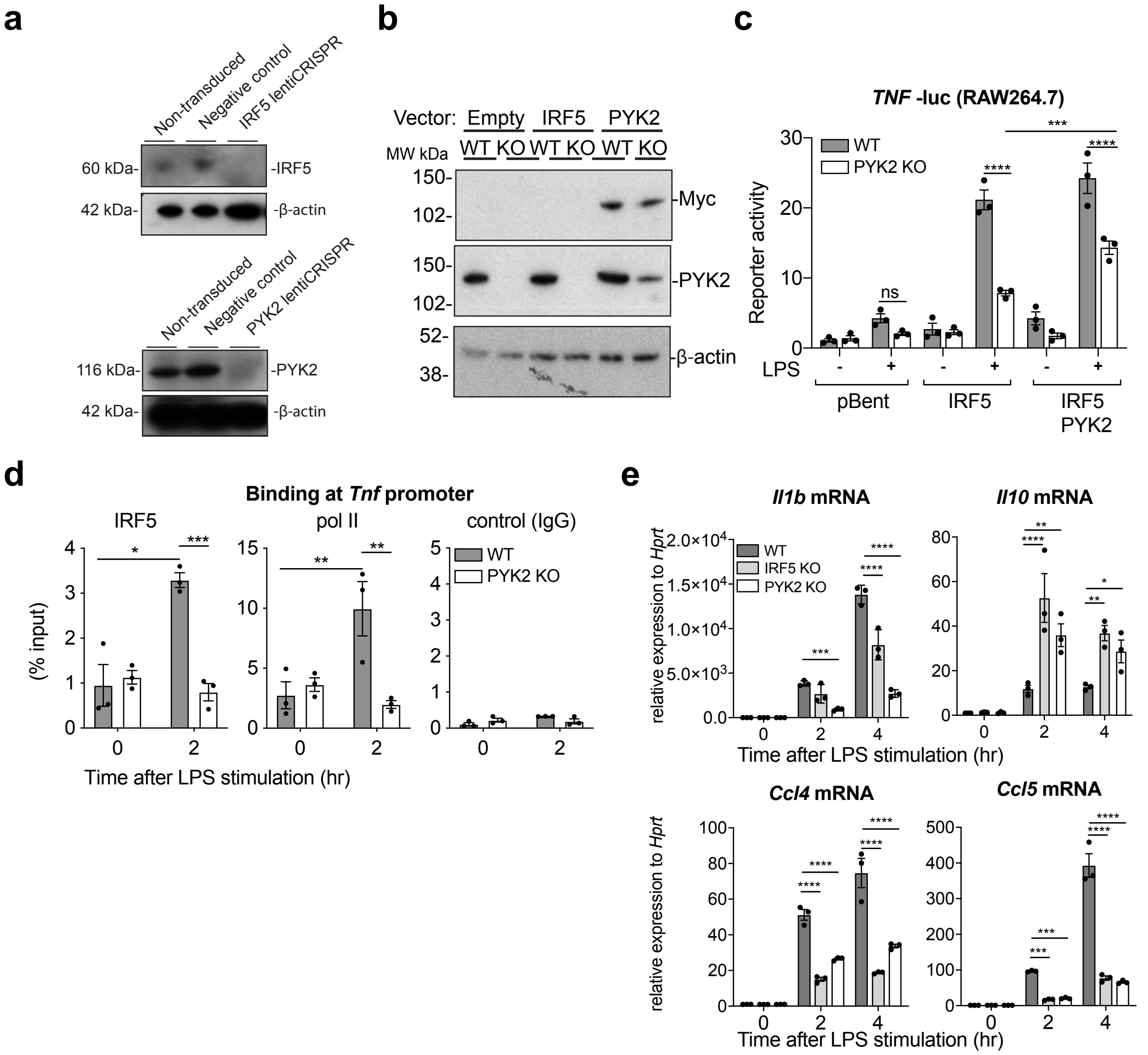

Supplementary Fig. 2. PYK2 knockout in RAW264.7 macrophages and IRF5 activation. (a) Western blot analysis of PYK2 and IRF5 expression in RAW 264.7 cells transfected with control, PYK2 or IRF5 CRISPR-based knockout constructs. **(b)** Immunoblot analysis for restoring PYK2 expression in PYK2 deficient RAW264.7 cells. Representative blots from three independent experiments are shown for (a-b). **(c)** TNF-luciferase activity in WT and PYK2 KO RAW264.7 cells co-transfected with pBent2-Empty, HA-IRF5, or Myc-PYK2 along with TNF-firefly Luc and pRLTK-Renilla Luc. Cells were stimulated with LPS (1 µg/ml) or left untreated for a further 6 hrs. **(d)** IRF5 and pol II binding to Tnf gene promoter in resting or LPS-treated (2h, 500 ng/ml) wild type or PYK2 KO RAW264.7 cells as measured by the chromatin immunoprecipitation (ChIP) method. A non-specific IgG antibody was used as a negative control for ChIP. Data are normalized against chromatin amount in lysates (and expressed as percentage of input for each gene). **(e)** Gene expression levels in wild type, PYK2 KO or IRF5 KO RAW264.7 cells stimulated with LPS (500 ng/ml) for 0, 2, or 4 hrs. Gene expression was measured by qPCR. All values in **(c-e)** are shown as mean values ± SEM from n=3 independent experiments. Statistical significance was calculated with two-way ANOVA with Sidak correction (*P<0.05, ** P<0.01, *** P<0.001, and **** P<0.0001). Source data are provided as Source Data file.

Supplementary Figure 3

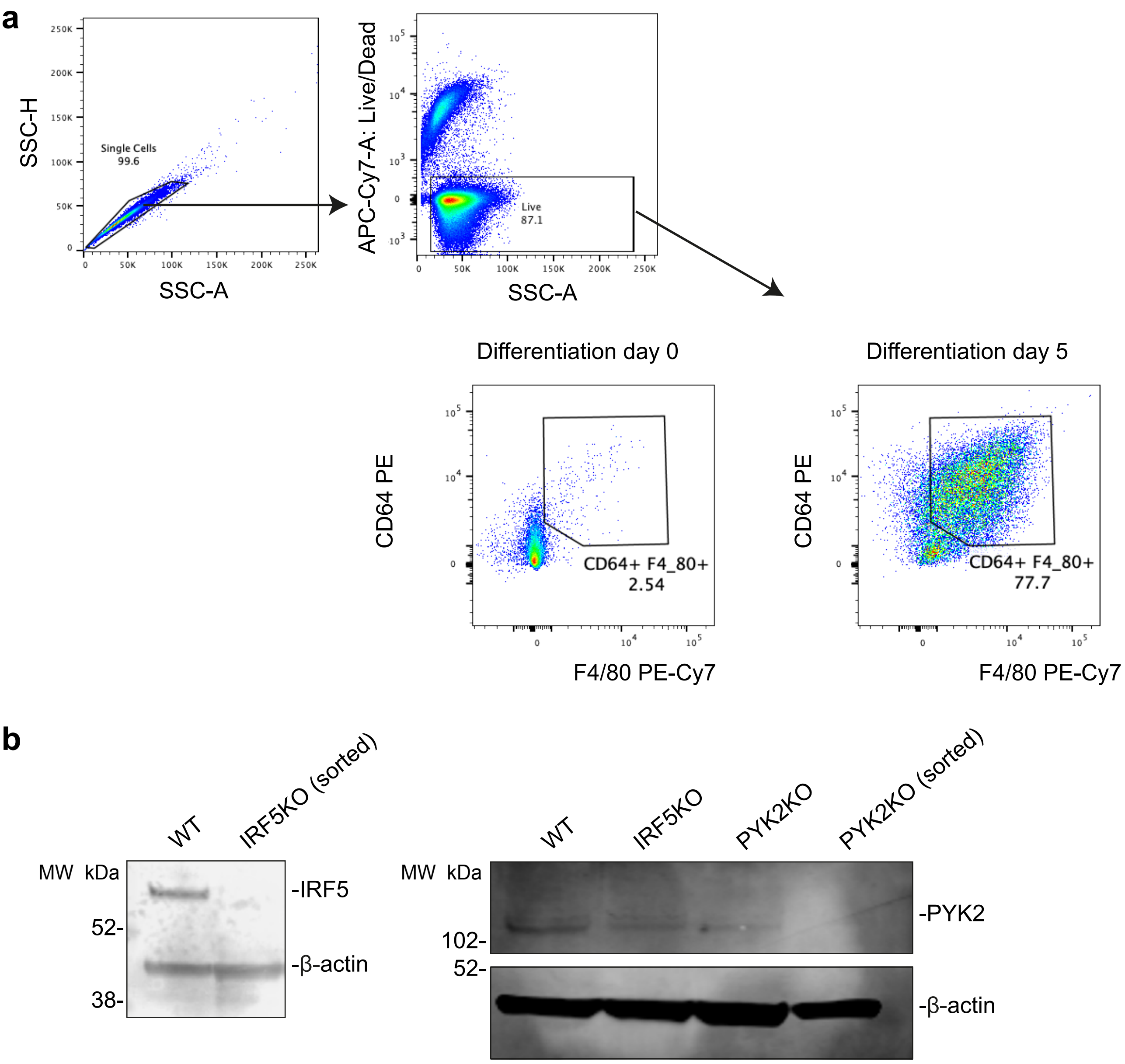

Supplementary Fig. 3. PYK2 knockout in HoxB8 macrophages. **(a)** Gating strategy to determine the percentage of differentiated Hoxb8 cells after 5 days with GM-CSF. Day 0 corresponds to cells prior to differentiation. **(b)** Western blot analysis of IRF5 and PYK2 expression in Hoxb8 macrophage progenitors transfected with PYK2 or IRF5 CRISPR-based knockout constructs. Representative blot from three independent experiments are shown. Source data are provided as Source Data file.

Supplementary Figure 4

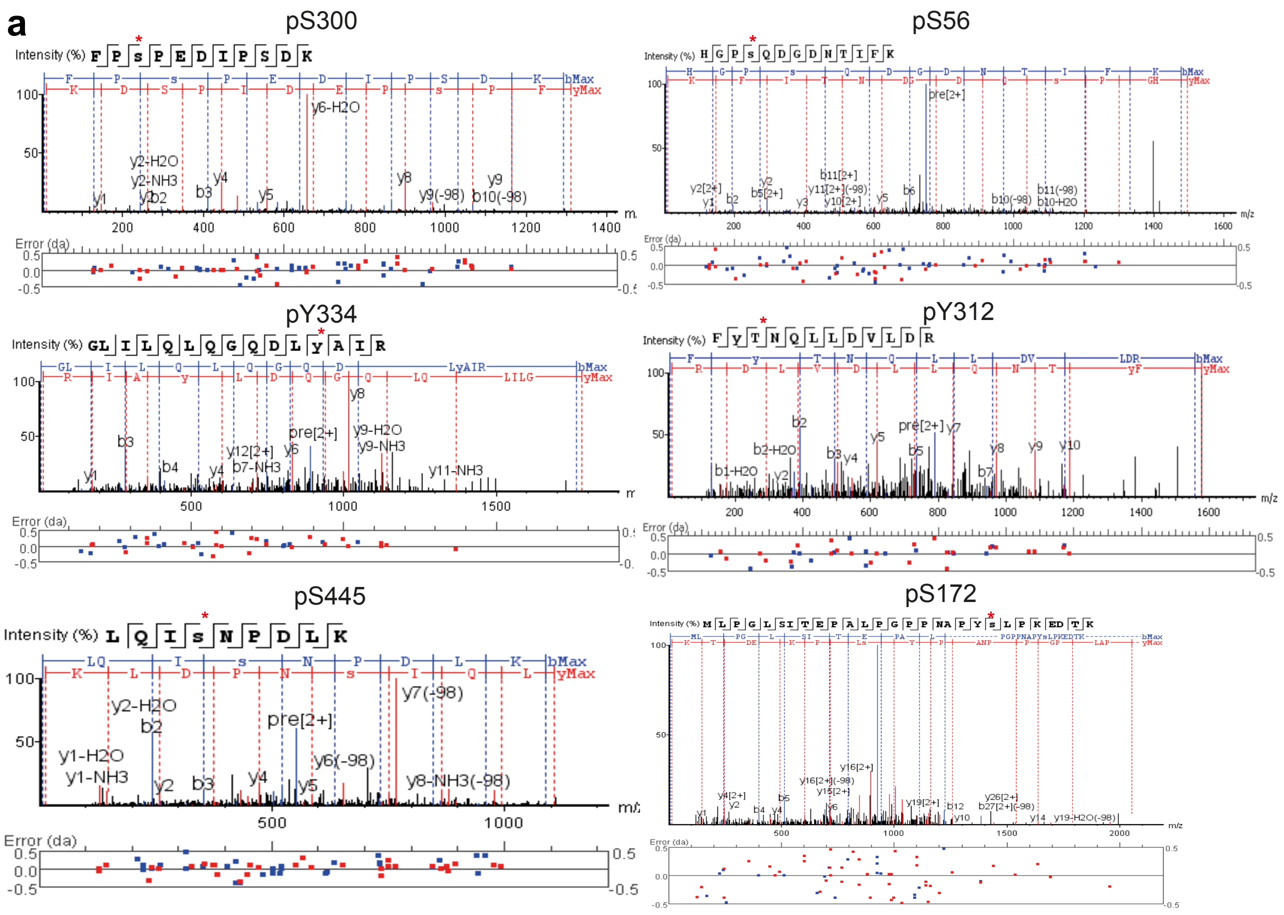

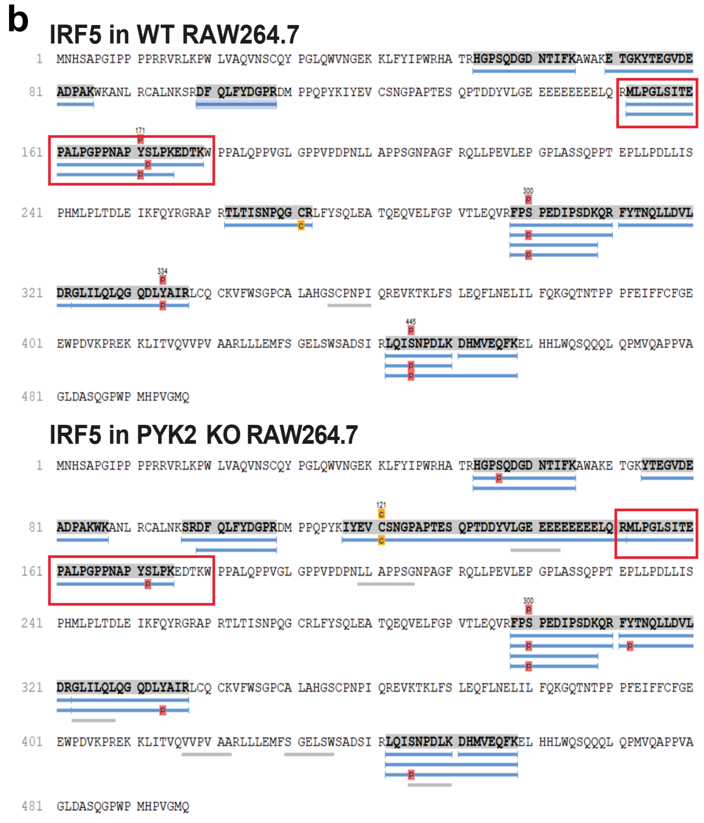

c

Mouse IRF5 site	Human IRF5-v2 site
Y104	Y104
Y171	Y172
S172	S173
Y312	Y329
Y334	Y351

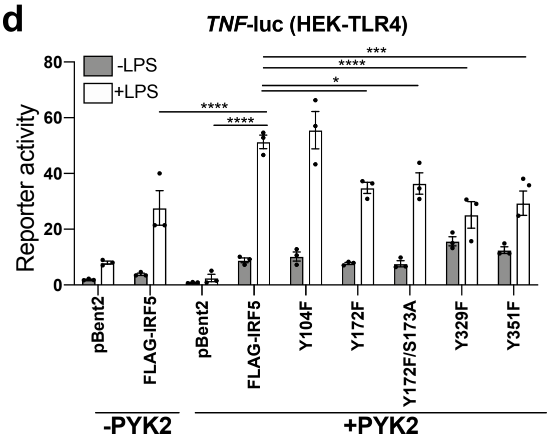

Supplementary Fig. 4. Mass spectrometry analysis detects distinct IRF5 phosphotylation sites in WT and PYK2 KO macrophages. (a) MS/MS spectra indicating mouse IRF5 S300, Y334, S445, S56, Y312, S172 phosphorylation in LPS-stimulated WT and PYK2 KO cells. Fragmentation ions of the b- and y- series are indicated in blue and red, respectively. **(b)** Identification of endogenous IRF5 phosphorylation sites by tandem mass spectrometry (MS/MS) in LPS-stimulated WT (top panel) and PYK2 KO (lower panel) RAW264.7 cells in which the peptides identified by LC-MS/MS are underlined in blue. Each line corresponds to a unique MS/MS spectrum. Post-translational modifications including cysteine carbamidomethylation

Supplementary Figure 5

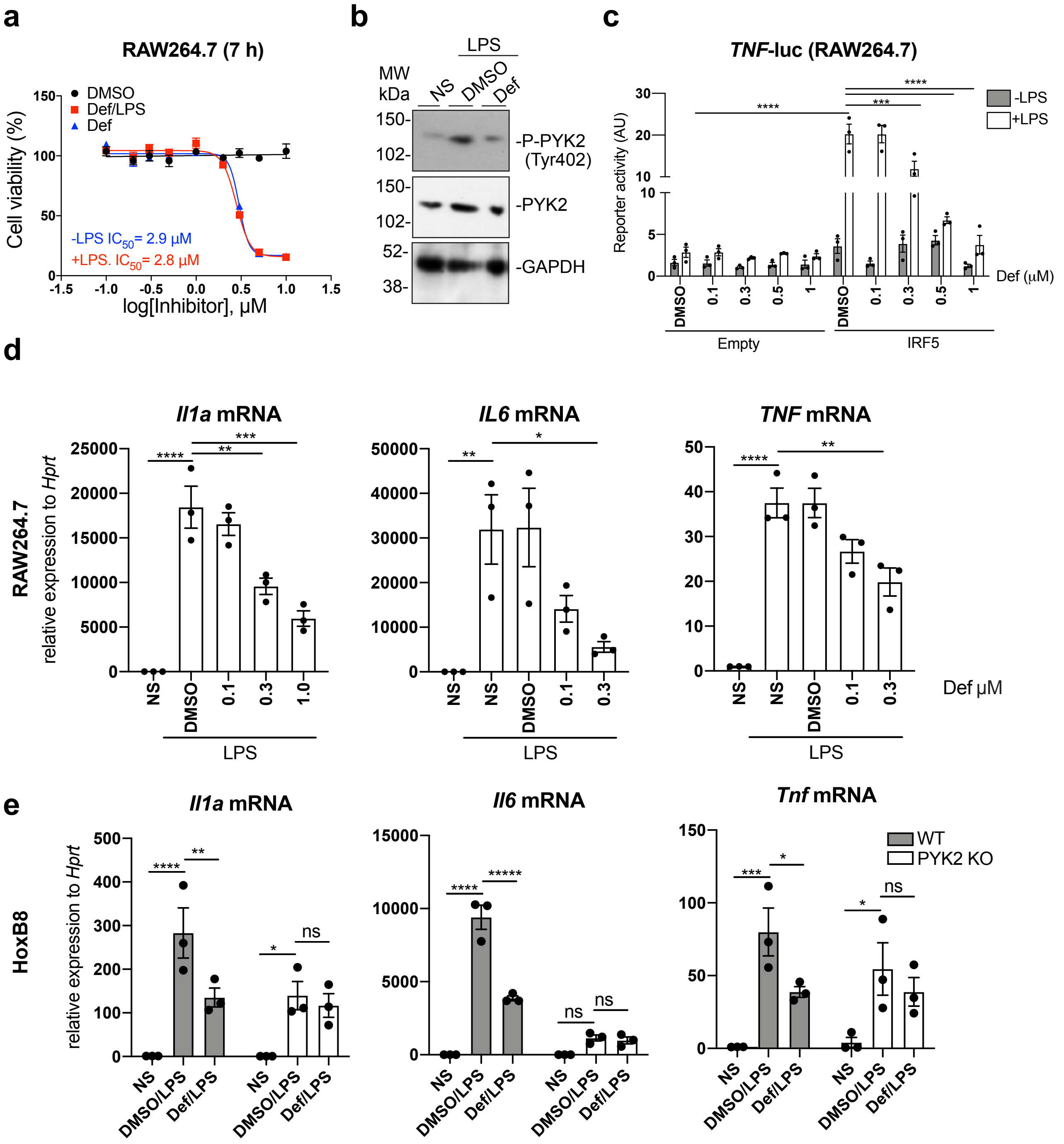

Supplementary Fig. 5. Defactinib inhibits PYK2 phosphorylation and IRF5-driven gene expression. **(a)** Cell viability in RAW264.7 cells pre-treated with DMSO/Defactinib for 1 hr followed by LPS (1µg/mL) for 6 hrs. IC50, inhibitor concentration at which 50% decline in cell viability was observed compared to control (DMSO). Data are presented as mean values ±SD from n=3 independent experiments. **(b)** Immunoblot of lysates of RAW264.7 cells pre-treated for 1 h with 1µM defactinib (def) or DMSO control, and stimulated with LPS (1µg/mL) for 30 min. Blots were probed with Abs specific for PYK2 phosphorylated on Tyr-402, total PYK2 and GAPDH. Representative blot from three independent experiments are shown. **(c)** TNF-luc reporter activity in the absence or presence of ectopically expressed IRF5 in RAW264.7 cells pre-treated for 1 hr with defactinib (or DMSO control) at indicated concentrations followed by LPS (1µg/ml) for 6 hrs. Data displayed as mean values ±SEM from n=3 independent experiments. Statistical significance was calculated by two-way ANOVA with Sidak correction (*** P<0.001 and ****P<0.0001). **(d)** Gene expression levels in RAW264.7 cell pre-treated with defactinib (def) or DMSO control for 1 h, followed by LPS stimulation for 4 hrs. Data displayed as mean values ±SEM from n=3 independent experiments and analysed by one-way ANOVA with Sidak correction (*P<0.05, ** P<0.01, ***P<0.001 and ****P<0.0001). **(e)** Gene expression levels in wild type and PYK2 KO HoxB8 macrophages pre-treated for 1 hr with defactinib (Def) or DMSO control and stimulated with LPS (100 ng/ml) for 2 hrs. Data shown as mean values ±SEM from n=3 independent experiments. Statistical significance was analysed by two-way ANOVA with Sidak's post-hoc test (*P<0.05, ** P<0.01, ***P<0.001 and ****P<0.0001). Source data are provided as Source Data file.

Supplementary Figure 6

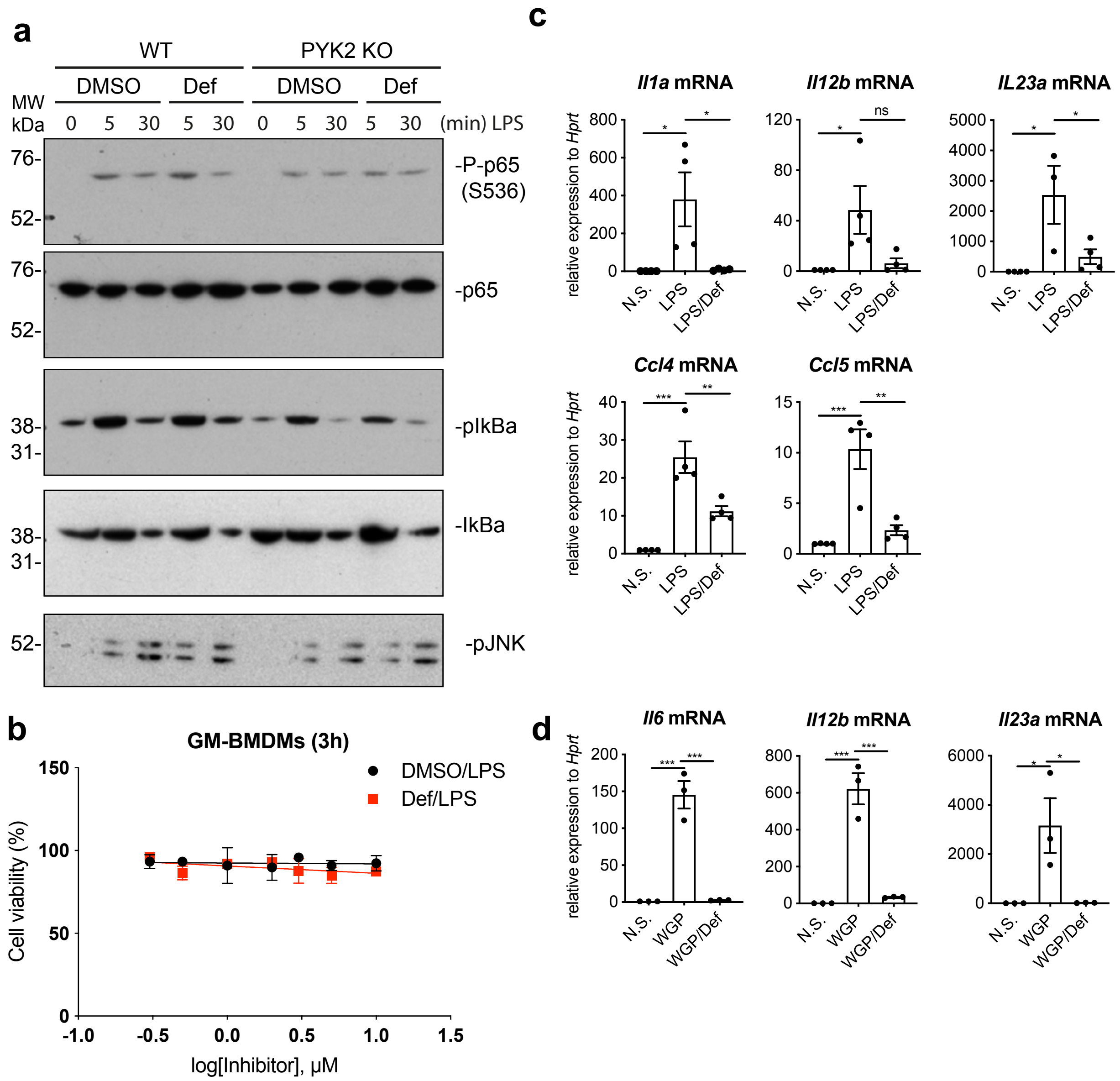

Supplementary Fig. 6. PYK2 deletion and inhibition has no effect on NFkB and JNK signalling (a) WT and PYK2 KO RAW264.7 cells were pre-treated with 1 μM of defactinib (Def) or DMSO vehicle control for 1 h followed by LPS (500 ng/ml) at indicated timepoints. Cell lysates were subjected for immunoblot using indicated antibodies. Representative blot from three independent experiments are shown. **(b)** Cell viability in GM-BMDMs pre-treated with DMSO/Defactinib for 1 hr followed by LPS (100 ng/ml) for 2 hrs. Error bars represent mean from 2 replicates. **(c)** Gene expression levels in GM-BMDMs pre-treated with 3.5 μM defactinib (def) or DMSO control for 1 h, followed by LPS (100 ng/ml) or **(d)** WGP (100 ug/ml) for 2 hrs. Data are shown as means ±SEM for n=3-4 indepedent experiments. Statistical significance was analysed by one-way ANOVA with Tukey's correction *P<0.05, **P<0.01, and ***P<0.001. Source data are provided as Source Data file.

Supplementary Figure 7

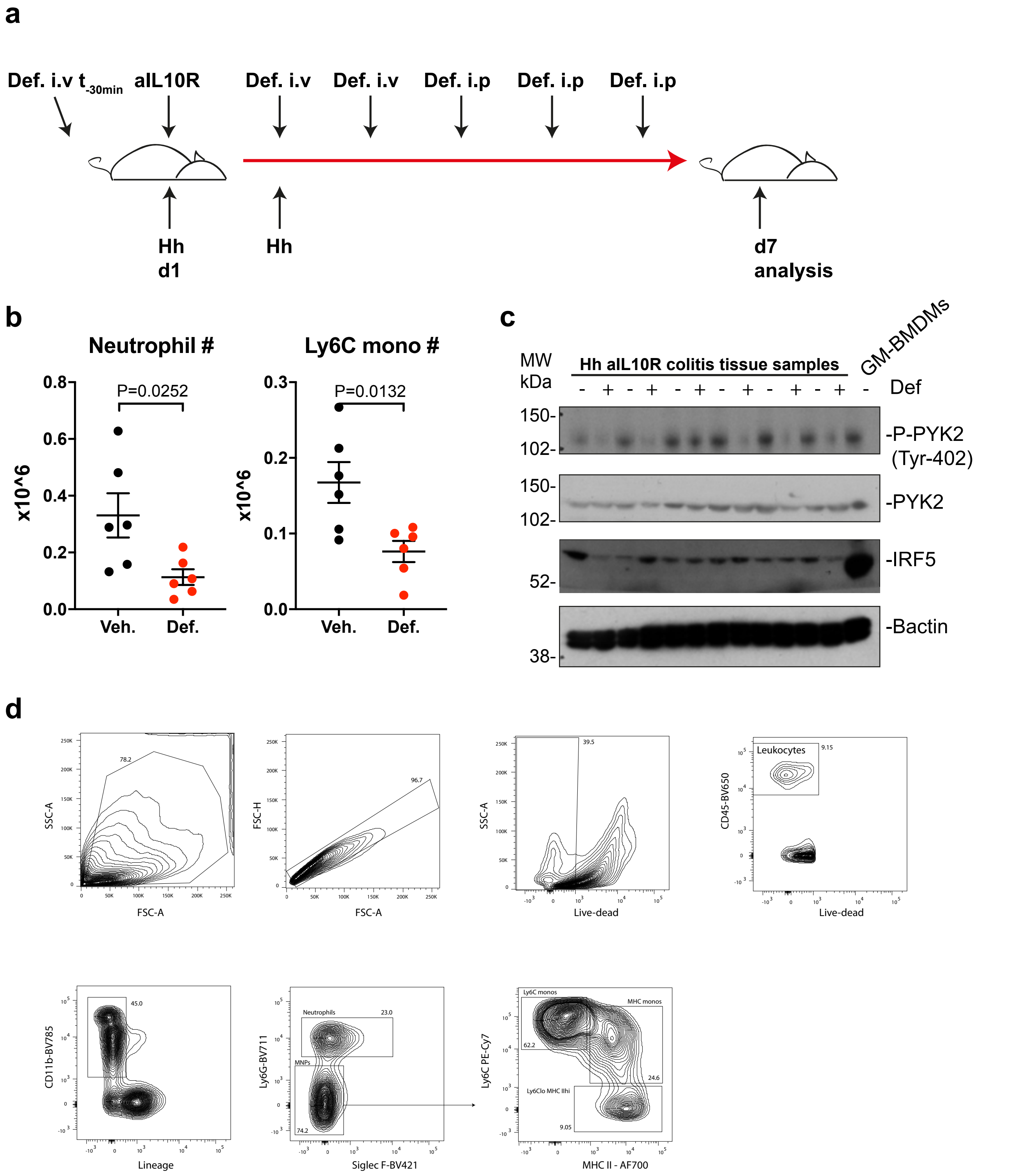

Supplementary Fig. 7. Defactinib in Hh/anti-IL10R-model of murine colitis. (a) Defactinib treatment regime during the initiation phase of mouse Hh+anti-IL-10R colitis. Diagram drawn by authors. **(b)** Immune cell infiltrate from Hh/antil-IL10R-treated mice, which received either vehicle or defactinib. Data shown as mean ±SEM from n=6 mice per condition. Statistical significance was calculated by a two-tailed unpaired t test. **(c)** PYK2 autophosphorylation (pY402) in vehicle or defactinib treated mice (n=6 mice per condition) assessed by western blot analysis. Lysates from LPS stimulated GM-BMDMs included as a positive control. **(d)** Gating strategy for (b). Source data are provided as Source Data file.

Supplementary Figure 8

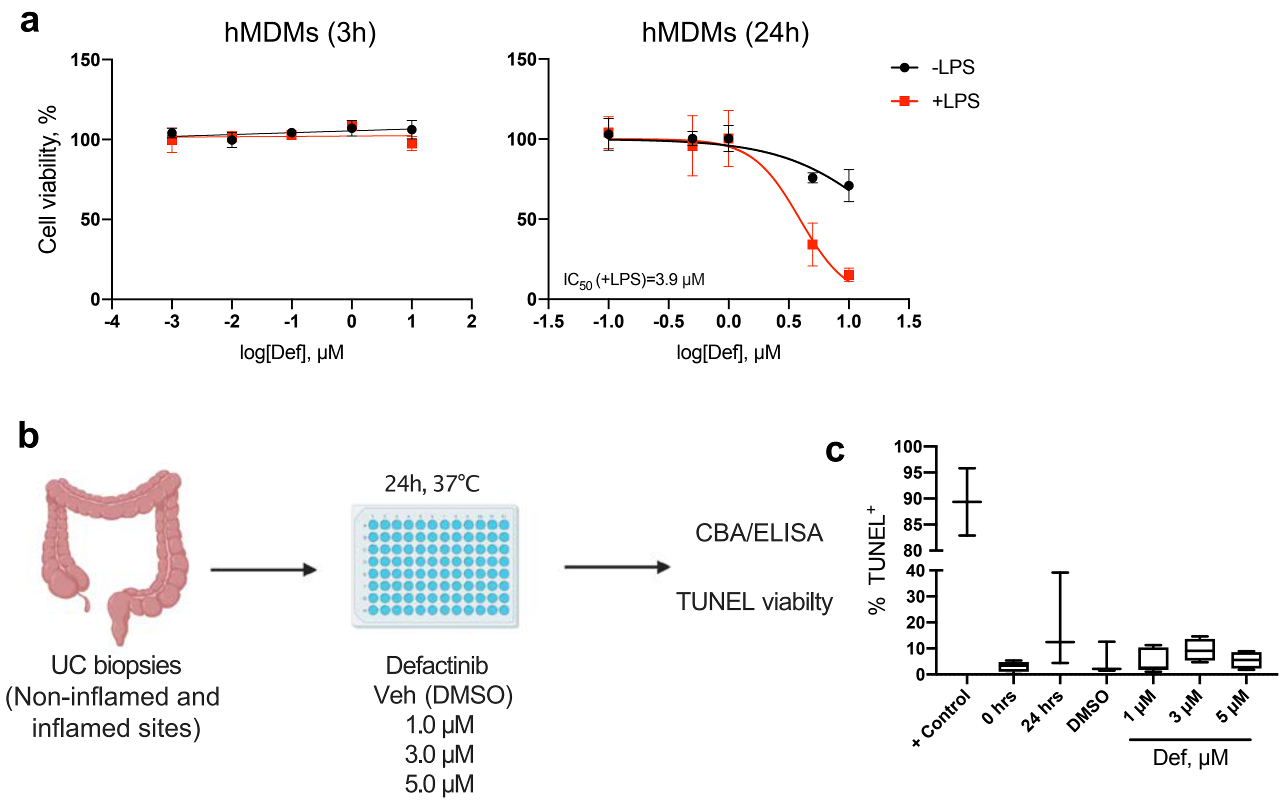

Supplementary Fig. 8. Defactinib in human monocyte-derived macrophages and UC biopsies. (a) Cell viability was measured in human monocyte-derived macrophages (hMDMs) after 3hrs or 24 hrs of treatment with defactinib. Data shown as mean ±SD from n=3 independent experiments. **(b)** Defactinib treatment of human biopsies from inflamed and non-inflamed sites of patients with ulcerative colitis. Diagram created with BioRender.com. **(c)** Cell viability by TUNEL assay was measured in colon biopsies after 24 hr treatment with defactinib. Box–and- whisker plots represent the median, interquartile range (IQR), and minimum and maximum values. Data generated from n=3-5 independent experiments. Source data are provided as Source Data file.

Supplementary Figure 9

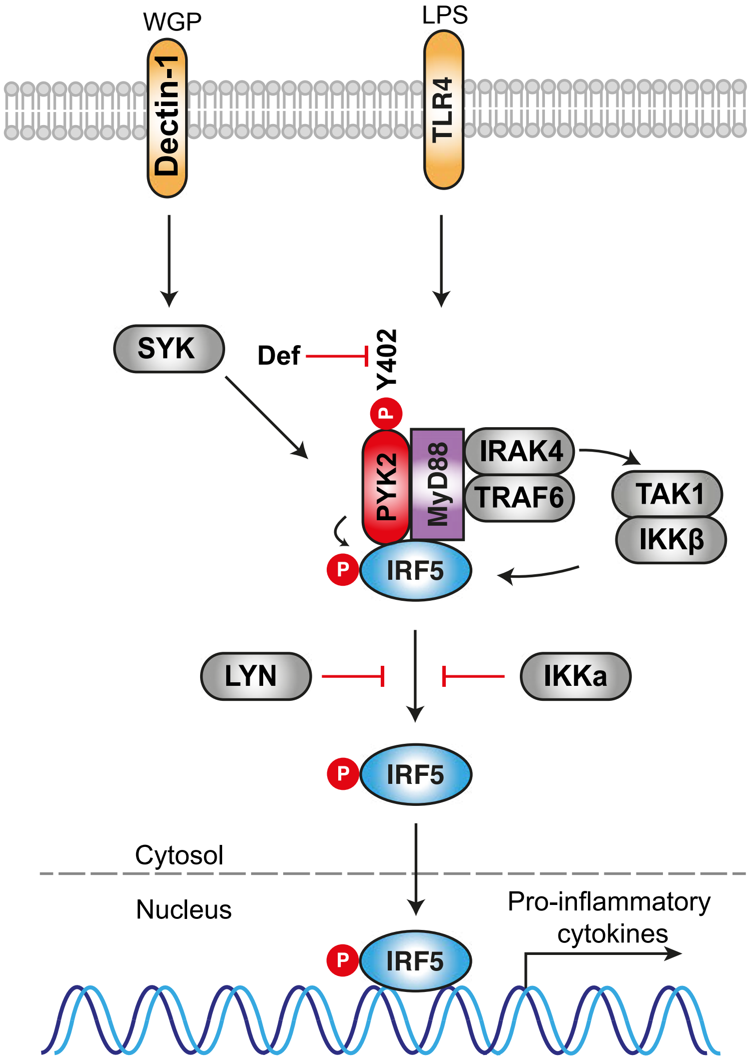

Supplementary Fig. 9. Proposed model of IRF5 activation by PYK2 in macrophages. LPS stimulation of TLR4 leads to PYK2 autophosphorylation on site Y402, which is inhibited by Defactinib. Activated PYK2 contributes to phosphorylation and activation of IRF5, which translocates to the nucleus and binds to target genes. Serine kinases IRAK4, TAK1, and IKKβ have been proposed to phosphorylate and activate IRF5 downstream of the TLR-MyD88 pathway[12–15], while IKKα and Lyn negatively regulated IRF5[19,67]. Dectin-1 stimulation by whole glucan particles also leads to IRF5 mediated transcription and is likely to be Syk-dependent.

Supplementary Table 1 List of probes used

Reagent type	Gene	Product code/sequence	Company
Mouse Taqman probe	Ccl4	Mm00443111_m1	Thermo Fisher
Mouse Taqman probe	Ccl5	Mm01302427_m1	Thermo Fisher
Mouse Taqman probe	Hprt	Mm03024075_m1	Thermo Fisher
Mouse Taqman probe	Il1a	Mm00439620_m1	Thermo Fisher
Mouse Taqman probe	Il1b	Mm00434228_m1	Thermo Fisher
Mouse Taqman probe	Il6	Mm00446190_m1	Thermo Fisher
Mouse Taqman probe	Il10	Mm00439614_m1	Thermo Fisher
Mouse Taqman probe	Il12b	Mm01288989_m1	Thermo Fisher
Mouse Taqman probe	Il23a	Mm00518984_m1	Thermo Fisher
Mouse Taqman probe	Tnf	Mm00443258_m1	Thermo Fisher
Human Taqman probe	IL1a	Hs00174092_m1	Thermo Fisher
Human Taqman probe	IL6	Hs00913644_m1	Thermo Fisher
Human Taqman probe	IL12a	Hs01073447_m1	Thermo Fisher
Human Taqman probe	IL12b	Hs01011518_m1	Thermo Fisher
Human Taqman probe	IL23a	Hs00372324_m1	Thermo Fisher
Human Taqman probe	RPLP0	Hs00420895_gH	Thermo Fisher
Human Taqman probe	TNF	Hs00174128_m1	Thermo Fisher
ChIP primers	Il1a	F-ACTTCTGGTGCTCATCTGTCATGTT<br>R-GCTCTATGGTTCCTGTGTCTGTAGG	Thermo Fisher
ChIP primers	Il1b	F-GGATGTGCGGAACAAAGGTAGGCACG<br>R-ACTCCAACTGCAAAGCTCCCTCAGC	Thermo Fisher
ChIP primers	Il6	F-GAGAGAGGAGTGTGAGGCAGAGAGC<br>R-GGTTGTCACCAGCATCAGTCCCAAG	Thermo Fisher
ChIP primers	Tnf	F-GCTAAGTTCTTCCCCATGGATGTCCC<br>R-ACCCATTTCTTCTCTGTCCTCCAGAGC	Thermo Fisher

Supplementary Table 2. List of antibodies used for western blots

Reagent type	Designation	Company	Catalog #	Dilution
Primary antibody	alpha-tubulin	CST	3873	1:1000
Primary antibody	beta-actin	Sigma	A5441	1:5000
Primary antibody	GAPDH	Abcam	AB9485	1:1000
Primary antibody	Histone H3	Abcam	AB1791	1:1000
Primary antibody	IkBa	CST	9242	1:1000
Primary antibody	IkBa (Phospo-Ser32)	CST	2859	1:500
Primary antibody	IRF5	Abcam	AB21689	1:1000
Primary antibody	c-Myc	SCB	sc-40	1:1000
Primary antibody	NFkB p65	SCB	sc-372	1:1000
Primary antibody	NFkB p65 (phospho S536)	Abcam	AB86299	1:1000
Primary antibody	PYK2	CST	3292	1:1000
Primary antibody	PYK2 (Phospho-Tyr402)	CST	3291	1:200
Primary antibody	Phospho-JNK	Abcam	AB4821	1:500
HRP-conjugated antibody	FLAG M2	Sigma	A8592	1:5000
HRP-conjugated antibody	HA	Roche	12013819001	1:5000
HRP-conjugated antibody	Strep	IBA	2-1509-001	1:5000
Secondary antibody	Mouse secondary	Dako	P0260	1:5000
Secondary antibody	Rabbit secondary	GE Healthcare	NA934	1:5000
Secondary antibody	Rabbit Trueblot secondary	Rockland	18-8816-31	1:5000

Supplementary Table 3. List of antibodies used for flow cytometry

Antigen	Colour	Clone	Manufacturer	Catalog #	Dilution
CD45	BV650	30-F11	Biolegend	103151	1:200
CD11b	BV785	M1/70	Biolegend	101243	1:200
CD11c	BV605	N418	Biolegend	117334	1:200
Siglec F	BV421	E50-2440	BD Bioscience	562681	1:200
Ly6G	BV711	1A8	Biolegend	127643	1:200
F4/80	PE-Dazzle594	BM8	Biolegend	123146	1:200
F4/80	PE-Cy7	BM8	Biolegend	123113	1:200
CD103	PE	2E7	BD Bioscience	557495	1:200
CD206	APC	CO682L	Biolegend	141707	1:200
MHC II	AF 700	M5/114.15.2	Invitrogen	56-5321-82	1:200
CD19	PerCP-Cy5.5	6D5	Biolegend	115533	1:200
CD138	PerCP-Cy5.5	281-2	Biolegend	142509	1:200
NK1.1	PerCP-Cy5.5	PK136	Biolegend	108727	1:200
CD3e	PerCP-Cy5.5	145-2C11	Biolegend	100327	1:200
TCRgd	PerCP-Cy5.5	GL3	Biolegend	118117	1:200
Ter119	PerCP-Cy5.5	TER-119	Biolegend	116227	1:200
CD64	PE	X54-5/7.1	Biolegend	139304	1:200
Lys6c	PE-Cy7	HK1.4	Biolegend	128017	1:200
IL10R blocking Ab	n/a	1B1.3A	2bscientific	BE0050	n/a
