# Supplementary material for: Defactinib inhibits PYK2 phosphorylation of IRF5 and reduces intestinal inflammation
Source: Nat Commun. 2021 Nov 18;12:6702. doi: 10.1038/s41467-021-27038-5 (PMC8602323; doi:10.1038/s41467-021-27038-5)
Supplement: Supplementary file 3 — Reporting Summary [file 41467_2021_27038_MOESM3_ESM.pdf]

nature research

Corresponding author(s): IRINA UDALOVA

Last updated by author(s): Sep 23, 2021

Reporting Summary

Nature Research wishes to improve the reproducibility of the work that we publish. This form provides structure for consistency and transparency in reporting. For further information on Nature Research policies, see Authors & Referees and the Editorial Policy Checklist .

Statistics

For all statistical analyses, confirm that the following items are present in the figure legend, table legend, main text, or Methods section.

n/a	Confirmed	
☐	☒	The exact sample size (n) for each experimental group/condition, given as a discrete number and unit of measurement
☐	☒	A statement on whether measurements were taken from distinct samples or whether the same sample was measured repeatedly
☐	☒	The statistical test(s) used AND whether they are one- or two-sided<br>*Only common tests should be described solely by name; describe more complex techniques in the Methods section.*
☒	☐	A description of all covariates tested
☐	☒	A description of any assumptions or corrections, such as tests of normality and adjustment for multiple comparisons
☐	☒	A full description of the statistical parameters including central tendency (e.g. means) or other basic estimates (e.g. regression coefficient) AND variation (e.g. standard deviation) or associated estimates of uncertainty (e.g. confidence intervals)
☐	☒	For null hypothesis testing, the test statistic (e.g. F, t, r) with confidence intervals, effect sizes, degrees of freedom and P value noted<br>*Give P values as exact values whenever suitable.*
☒	☐	For Bayesian analysis, information on the choice of priors and Markov chain Monte Carlo settings
☒	☐	For hierarchical and complex designs, identification of the appropriate level for tests and full reporting of outcomes
☒	☐	Estimates of effect sizes (e.g. Cohen's d, Pearson's r), indicating how they were calculated

Our web collection on statistics for biologists contains articles on many of the points above.

Software and code

Policy information about availability of computer code

Data collection	Real-time PCR reactions were performed on a ViiA7 system (Life Technologies)<br>Luminescent levels were recorded by the BMG FLUOstar Omega microplate reader (BMG Labtech) on the MARS Data Analysis software (V4.00 R2). ELISAs were measured on the SPECTROstar Omega microplate reader (BMG Labtech) on the MARS Data Analysis software (V4.00 R2). Diva 8.0.1 (BD Biosciences) was used to collect FACS data
Data analysis	Prism (v8.0) was used to generate all graphs and statistic with the exception of RNA-seq. Differential expression was analysed with DESeq2. Gene set enrichment analysis was performed using one-sided Fisher's exact tests (as implemented in the 'gsfisher' R package https://github.com/sansomlab/gsfisher/).Raw MS files were subjected to processing using PEAKS (version 8.5) software and searched against the UniProtSP (P56477) Mus Musculus database. Flowjo (v10.5.1, Tree Star) was used for all flow cytometry analysis.

For manuscripts utilizing custom algorithms or software that are central to the research but not yet described in published literature, software must be made available to editors/reviewers. We strongly encourage code deposition in a community repository (e.g. GitHub). See the Nature Research guidelines for submitting code & software for further information.

Data

Policy information about availability of data

All manuscripts must include a data availability statement. This statement should provide the following information, where applicable:

- Accession codes, unique identifiers, or web links for publicly available datasets
- A list of figures that have associated raw data
- A description of any restrictions on data availability

MS raw files have been deposited to Proteome Xchange Consortium via the PRIDE (53) partner repository with the dataset identifier PXD014033 (https://

www.ebi.ac.uk/pride/archive/). These data are associated with Figures 3a-b and Supplementary figures 4a-b. RNA sequencing data associated with Figures 5a-e and have been deposited in GEO with the accession code GSE141082 (Token code available on request).

Field-specific reporting

Please select the one below that is the best fit for your research. If you are not sure, read the appropriate sections before making your selection.

- [x] Life sciences
- [ ] Behavioural & social sciences
- [ ] Ecological, evolutionary & environmental sciences

For a reference copy of the document with all sections, see nature.com/documents/nr-reporting-summary-flat.pdf

Life sciences study design

All studies must disclose on these points even when the disclosure is negative.

Sample size

Animal sample size estimates were determined using power analysis (power=90% and alpha = 0.05) based on the mean and standard deviation from our previous studies and/or pilot studies using 6 animals per group. Sample size of n=3 biological replicates (each in technical replicates) was chosen as a suitable sample size for cell culture as repeats gave similar results.

Data exclusions

No samples were excluded

Replication

All attempts at replication were successful. Results shown are the result of three or more independent experiments. N number describes biological replicates such as mice and cells.

Randomization

Animals from the same cage were randomly selected for different treatment.

Blinding

Pathology analysis was blinded during data collection. For all other analyses, blinding was not performed as the experimental endpoints and readouts are quantitative and not subjective.

Reporting for specific materials, systems and methods

We require information from authors about some types of materials, experimental systems and methods used in many studies. Here, indicate whether each material, system or method listed is relevant to your study. If you are not sure if a list item applies to your research, read the appropriate section before selecting a response.

Materials & experimental systems

n/a	Involved in the study	
	x	Antibodies
	x	Eukaryotic cell lines
x		Palaeontology
	x	Animals and other organisms
	x	Human research participants
x		Clinical data

Methods

n/a	Involved in the study	
x		ChIP-seq
	x	Flow cytometry
x		MRI-based neuroimaging

Antibodies

Antibodies used

Anti-alpha-tubulin, CST, 3873, 1:1000
Anti-beta-actin, Sigma, A5441 1:5000
Anti-GAPDH, Abcam, AB9485 1:1000
Anti- Histone H3, Abcam, AB1791 1:1000
Anti-IkBa, CST, 9242 1:1000
Anti-IkBa (Phospo-Ser32), CST, 2859, 1:500
Anti-IRF5 Abcam, AB21689, 1:1000
Anti-c-Myc, SCB, sc-40, 1:1000
Anti-NFkB p65, SCB, sc-372, 1:1000
Anti-NFkB p65 (phospho S536), Abcam, AB86299, 1:1000
Anti-PYK2, CST, 3292, 1:1000
Anti-PYK2 (Phospho-Tyr402), CST, 3291, 1:200
Anti-Phospho-JNK, Abcam, AB4821, 1:500
Anti-FLAG M2-Peroxidase (HRP), Sigma, A8592, 1:5000
Anti-HA (HRP), Roche, 12013819001 1:5000
Anti-Strep (HRP), IBA, 2-1509-001, 1:5000
Anti-Mouse IgG secondary antibody, Dako, P0260, 1:5000

Anti-Rabbit IgG secondary antibody, GE Healthcare, NA934, 1:5000
Trueblot Anti-Rabbit IgG secondary antibody, Rockland, 18-8816-31, 1:5000
CD45, BV650, 30-F11, Biolegend, 103151, 1:200
CD11b, BV785, M1/70, Biolegend, 101243 1:200
CD11c BV605 N418 Biolegend 117334 1:200
Siglec F BV421 E50-2440 BD Bioscience 562681 1:200
Ly6G BV711 1A8 Biolegend 127643 1:200
F4/80 PE-Dazzle594 BM8 Biolegend 123146 1:200
F4/80 PE BM8 Biolegend 123113 1:200
CD103 PE 2E7 BD Bioscience 557495 1:200
CD206 APC CO68L2 Biolegend 141707 1:200
MHC II AF 700 M5/114.15.2 Invitrogen 56-5321-82 1:200
CD19 PerCP-Cy5.5 6D5 Biolegend 115533 1:200
CD138 PerCP-Cy5.5 281-2 Biolegend 142509 1:200
NK1.1 PerCP-Cy5.5 PK136 Biolegend 108727 1:200
CD3e PerCP-Cy5.5 145-2C11 Biolegend 100327 1:200
TCRgd PerCP-Cy5.5 GL3 Biolegend 118117 1:200
Ter119 PerCP-Cy5.5 TER-119 Biolegend 116227 1:200
CD64, PE, X54-5/7.1 Biolegend, 139304, 1:200
Lys6c, PE-Cy7, HK1.4, Biolegend, 128017, 1:200
For in vivo injections, IL-10R blocking antibody (clone 1B1.2), 2bscientific, BE0050

Validation

All antibodies used for western blotting were purchased from commercial vendors. Validation of antibodies used in current study is described in technical data sheets provided by manufacturers websites.

All antibodies used for flow cytometry are well-established lineage and cell surface marker antibodies. Staining patterns were consistent with the manufacturer product information as well as published results.

Eukaryotic cell lines

Policy information about cell lines

Cell line source(s)

All cell lines (RAW264.7, HEK293-TLR4-CD14/Md2 and HEK293-ET cells) used in this study were purchased from InvivoGen

Authentication

Cell lines were validated by morphology and surface marker phenotype

Mycoplasma contamination

All cell lines tested negative for Mycoplasma contamination

Commonly misidentified lines
(See ICLAC register)

No misidentified lines were used

Animals and other organisms

Policy information about studies involving animals; ARRIVE guidelines recommended for reporting animal research

Laboratory animals

C57Bl/6 mice were purchased from the University of Oxford BMS. Male C57BL/6J mice aged 8-11 weeks were used for the experiment. Experimental groups consisted of 5-7 co-housed mice and were blinded to the researchers. Mice were bred and maintained under SPF conditions in accredited animal facilities at the University of Oxford. Animals were housed in individually ventilated cages at a constant temperature (20-23.3 degree Celsius) with 12h dark/light cycle, supplied with with food and water ad libitum.

Wild animals

No wild animals were used

Field-collected samples

This study does not use wild animals

Ethics oversight

All procedures were conducted according to the Operations of Animals in Scientific Procedures Act (ASPA) of 1986 and approved by the Kennedy Institute of Rheumatology Ethics Committee.

Note that full information on the approval of the study protocol must also be provided in the manuscript.

Human research participants

Policy information about studies involving human research participants

Population characteristics

Intestinal pinch biopsies were obtained from patients with Ulcerative Colitis registered in the Oxford IBD Cohort, attending the John Radcliffe Hospital Gastroenterology Unit (Oxford, UK). This cohort comprises 1896 patients with UC, median age 31 at

	diagnosis, treated with biological therapy (23%) or conventional steroids/immunomodulators (77%) for active disease, in addition to mesalazine.
Recruitment	Biopsies were collected during routine endoscopy. Informed, written consent was obtained from all donors
Ethics oversight	Human experimental protocols were approved by the NHS Research Ethics System (Reference numbers: 16/YH/0247)

Note that full information on the approval of the study protocol must also be provided in the manuscript.

Flow Cytometry

Plots

Confirm that:

- [x] The axis labels state the marker and fluorochrome used (e.g. CD4-FITC).
- [x] The axis scales are clearly visible. Include numbers along axes only for bottom left plot of group (a 'group' is an analysis of identical markers).
- [x] All plots are contour plots with outliers or pseudocolor plots.
- [x] A numerical value for number of cells or percentage (with statistics) is provided.

Methodology

Sample preparation	Colons and/or caeca were harvested from mice, washed in PBS/BSA and content flushed with forceps. Intestines were then opened longitudinally and washed once more before blotting to remove mucus. Gut tissue was then cut into 1 cm long pieces and placed in 50 mL centrifuge tube (Greiner) in ice cold PBS + 0.1% BSA. Colons were incubated 2 times at 300xg in 40 mL HBSS + 0.1% BSA + 1% Penicillin-Streptomycin (PS, Lonza) + 5mM EDTA (Sigma-Aldrich) at 37 °C for 10 min before the supernatant was aspirated. Tissue was placed in 40 mL PBS + 0.1% BSA + 1% PS for 5 min. Intestines were then incubated with 20 mL RPMI + 10% FCS +1% PS + 2.5 U/mL Collagenase VIII (Sigma-Aldrich) + 2 U/mL DNAse I (Roche), shaking for 45 mins - 1 hour at 37 °C. Supernatant was filtered through a 70 µm cell strainer to which 30 mL of ice cold PBS + 0.1% BSA + 1% PS + 5 mM EDTA was added to ablate collagenase/DNase activity. Cells were washed in 30 mL PBS/BSA before filtering once more through a 40 µm cell strainer. The cells were then pelleted by centrifugation at 400 rcf for 10 minutes at 4 °C and resuspended in 1 mL RPMI + 10% FCS + 1% PS before counting. Detailed information is provided in the methods section within the manuscript.
Instrument	CBA quantification of cytokine levels were performed on a FACSCanto II (BD). Aquisition of mouse samples was performed using either LSR II or Fortessa X20 flow cytometers
Software	Data were acquired using FACSDIVA V8.0.1 (BD). Data were analysed using Flowjo V10.5.1 (Treestar Inc.)
Cell population abundance	All sorted samples were checked for after-sorting purity (>98%)
Gating strategy	After debris (FSC-A vs SSC-A) and doublet (FSC-A vs FSC-H) exclusion, cells were gated Live CD45+ CD11b+ Dump-. The gating strategy provided in Supplementary Figure 7d was used to analyse myeloid cells in the colonic lamina propria of mice at day 7 of Helicobacter hepaticus + anti-IL10R colitis treated with either vehicle or defactinib. Positive populations were determined by FMO controls.

- [x] Tick this box to confirm that a figure exemplifying the gating strategy is provided in the Supplementary Information.
