# Supplementary material for: Defactinib inhibits PYK2 phosphorylation of IRF5 and reduces intestinal inflammation
Source: Nat Commun. 2021 Nov 18;12:6702. doi: 10.1038/s41467-021-27038-5 (PMC8602323; doi:10.1038/s41467-021-27038-5)
Supplement: Supplementary file 4 — Description of Additional Supplementary Files [file 41467_2021_27038_MOESM4_ESM.pdf]

Description of Additional Supplementary Files

File Name: Supplementary Data 1

Description: Reporter gene assay-based screening of the GSK PKIS set for inhibitors of IRF5 activation.
